# One-Year Outcomes of 1 Dose versus 3 Loading Doses Followed by Pro Re Nata Regimen Using Ranibizumab for Neovascular Age-Related Macular Degeneration: The ARTIS Trial

**DOI:** 10.1155/2019/7530458

**Published:** 2019-10-10

**Authors:** Fenghua Wang, Yuanzhi Yuan, Ling Wang, Xiaofeng Ye, Jingke Zhao, Mengxi Shen, Qi Zhang, Ding Xu, Guoyou Qin, Wei Zhang, Fei Yuan, Qing Chang, Peiquan Zhao, Fang Wang, Xiaodong Sun

**Affiliations:** ^1^Department of Ophthalmology, Shanghai General Hospital (Shanghai First People's Hospital), Shanghai Jiao Tong University, School of Medicine, Shanghai, China; ^2^Shanghai Engineering Center for Visual Science and Photomedicine, Shanghai, China; ^3^Department of Ophthalmology, Zhongshan Hospital, Fudan University, Shanghai, China; ^4^Department of Ophthalmology, Fudan University Eye and ENT Hospital, Fudan University, Shanghai, China; ^5^Department of Ophthalmology, Xinhua Hospital, Shanghai Jiao Tong University, School of Medicine, Shanghai, China; ^6^Department of Ophthalmology, Shanghai Tenth People's Hospital, Tongji University, School of Medicine, Shanghai, China; ^7^Department of Biostatistics, School of Public Health and Key Laboratory of Public Health Safety, Fudan University, Shanghai, China; ^8^Shanghai Key Laboratory of Visual Impairment and Restoration, Fudan University Eye and ENT Hospital, Fudan University, Shanghai, China; ^9^Shanghai Key Laboratory of Fundus Diseases, Shanghai, China

## Abstract

*Purpose*. To compare the functional and anatomical outcomes of one dose and three loading doses followed by the pro re nata (PRN) regimen in Chinese neovascular age-related macular degeneration (nvAMD) (including polypoidal choroidal vasculopathy (PCV)) patients. *Methods*. In this multicenter, prospective, open-label, controlled, 12-month study (ClinicalTrials.gov: NCT02810808), patients were randomized (1 : 1) to 1 dose + PRN (PRN group) or 3 loading doses + PRN (LD group) using intravitreal ranibizumab treatment. Best-corrected visual acuity (BCVA) and central retinal thickness (CRT) were evaluated. The main outcome was the change in BCVA. The noninferiority limit was 5 letters. *Results*. Forty-five patients in the PRN group and 49 patients in the LD group finished 12-month follow-up. Each group included 4 PCV patients. The mean change in BCVA from baseline was 7.8 letters in the PRN group, compared with 10.9 letters in the LD group (*P*=0.344). There were no significant differences between two groups in the mean change of CRT (−159.3 *μ*m vs. −120.5 *μ*m) at month 12. The mean number of injections during the 12-month follow-up was 6.0 in the PRN group and 6.8 in the LD group. The proportion of patients who gained an improvement in visual acuity by 15 or more letters was 28.9% in the PRN group and 44.9% in the LD group (*P*=0.066). *Conclusion*. One dose + PRN showed noninferior visual gains than 3 loading doses + PRN regimen using ranibizumab in Chinese nvAMD and PCV patients. Number of injections in the PRN group was similar as that in the LD group but remained a potential risk of vision instability during one-year follow-up using OCT-guided retreatment criteria. This trial is registered with NCT02810808.

## 1. Introduction

Neovascular age-related macular degeneration (nvAMD) is the fast-growing leading cause of blindness in China among aging population [1–3]. Since antivascular endothelial growth factor (anti-VEGF) agent ranibizumab (LUCENTIS®) has been launched in China in 2012, it has become the standard-of-care treatment of nvAMD [4].

MARINA and ANCHOR study [5, 6] proved that monthly intravitreal injections of ranibizumab can improve the mean best corrected visual acuity (BCVA). Pro re nata (PRN) regimen after three consecutive monthly loading doses (LD) using optical coherence tomography- (OCT-) guided evaluation has been used widely based on the results of HARBOR and PrONTO study [7, 8]. The Chinese Ocular Fundus Diseases society also published the panel advice in Clinical Pathway of Age-Related Macular Degeneration in China [9], which suggested the use of ranibizumab as a loading dose consisting of three initial consecutive injections at monthly intervals followed by OCT-guided retreatment. However, ranibizumab was of high cost and noninsurance covered in China. There were only 14% nvAMD patients receiving LD treatment, even in Shanghai, a highly developed city (2017 survey, unpublished data).

In the CATT study [10], patients in the PRN regimen without initial three-monthly intravitreal injections (as need) also showed statistically noninferior mean letters of BCVA compared with the patients receiving the monthly dosing ranibizumab regimen at one-year analysis. It will be interesting to know how this regimen works in Chinese nvAMD patients since 24.5% of them present with polypoidal choroidal vasculopathy (PCV) [11], a much higher prevalence than in Caucasian patients of 9.1% [12].

The ARTIS study was designed to compare the 12-month efficacy and safety of ranibizumab 0.5 mg administered PRN with and without the initial LD regimen in Chinese patients with nvAMD and PCV.

## 2. Methods

### 2.1. Study Design

This was a 12-month, prospective, multicenter, randomized, active treatment-controlled study conducted in 5 clinical sites in Shanghai, China. The study protocol was approved by all institutional Local Ethics review boards and was carried out in accordance with the declaration of Helsinki. Each patient provided a written informed consent before any study-related procedure. The study protocol was registered on www.ClinicalTrials.gov (NCT02810808).

Patients were eligible for the ARTIS study if they were ≥50 years old, diagnosed as nvAMD or PCV and treatment naïve. In the cases suspicious of PCV, we further performed indocyanine green angiography (ICGA) to confirm the diagnosis. Key exclusion criteria included the history of cataract surgery within 3 months or an arrangement of cataract surgery in the next 6 months from baseline, a history of any other intraocular surgeries, verteporfin photodynamic therapy (PDT), focal laser photocoagulation, transpupillary thermotherapy, previous treatment with antiangiogenic drugs, anecortave acetate, corticosteroids, etc., recent intraocular inflammation in the study eye, and intraocular pressure >30 mmHg.

### 2.2. Study Assessments

Each patient received an assessment of BCVA using standard ETDRS charts and an ophthalmic examination, including slit-lamp biomicroscopy, intraocular pressure, fundus biomicroscopy, fundus photography, and spectral-domain OCT at baseline and every visit point. Fluorescein angiography (FA) was performed at baseline and months 3 and 12. The OCT imaging, fundus photos, and FA results were evaluated by Shanghai Jiaotong University Eye Institute Reading Center (SJTURC).

Patients in the LD group were scheduled to have three-monthly intravitreal injections of ranibizumab 0.5 mg from month 0 to month 2. Patients in the PRN group were scheduled to receive one intravitreal injection at month 0. Then, both groups were followed up every month until month 12. In the PRN phase, retreatment criteria were referred to Chinese Ocular Fundus Diseases Society in Clinical Pathway of Age-related Macular Degeneration in China: “Signs of active neovascularization were defined as fluid on OCT (intraretinal fluid, subretinal fluid, or an enlargement of a pigment epithelial detachment), new or persistent hemorrhage, decreased visual acuity as compared with the previous examination, dye leakage, or increased lesion size on FA” [9]. The injection will be given within 3 days when the patient met the retreatment criteria.

### 2.3. Outcome Measures

The primary outcome was the mean change in BCVA between baseline and 1 year. Secondary outcomes included the proportion of patients with a gain or loss of 15 letters or more in BCVA, the mean change in central retinal thickness (CRT) on OCT, and the number of ranibizumab injections within one year. CRT was defined as the mean retinal thickness within the 1 mm diameter circle centered at the fovea. Total safety assessment was the incidence of ocular and systemic adverse events, including study eye serious adverse events (SAEs), arterial thromboembolic events (ATEs), and any adverse events potentially related to systemic VEGF-A inhibition.

### 2.4. Statistical Analysis

The noninferiority limit for the difference between two groups in the mean change of visual acuity at 1 year was 5 letters on the EDTRS chart [10]. Means were compared using independent-sample *t* tests and paired *t* tests. Percentages were compared using Pearson's *χ*^2^ tests. Statistical significance was defined as *P* < 0.05. All analyses were conducted using SPSS statistical software version 22.0 (IBM Corp., Armonk, NY).

## 3. Results

A total of 108 patients were included and randomized (1 : 1) to the LD group and PRN group in 5 clinical centers in Shanghai. Among them, visual scores were available for 45 of 54 patients (83%) in the PRN group and 49 of 54 (91%) in the LD group. Demographic and baseline characteristics are summarized in Table 1.

Baseline patient characteristics, including the mean age, BCVA letters, and CRT, were comparable between the PRN group and the LD group. Subtypes of AMD included 41 nvAMD eyes and 4 PCV eyes in the PRN group and 45 nvAMD eyes and 4 PCV eyes in the LD group.

### 3.1. Primary Outcome

**Figure 1:**
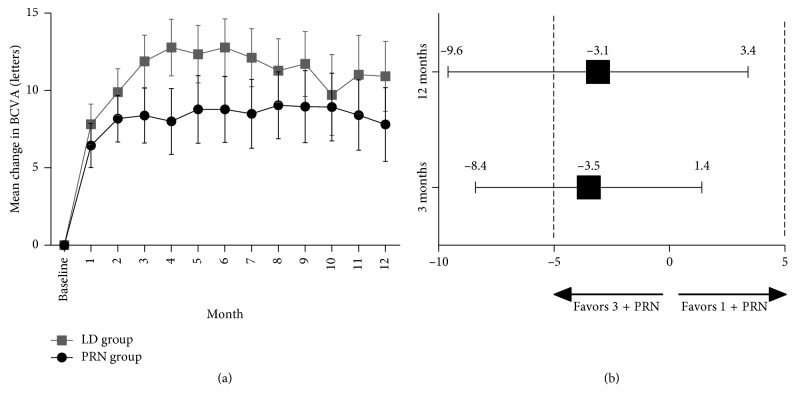
(a) Time course of best-corrected visual acuity (BCVA); (b) difference in mean change in BCVA at month 3 and month 12 between the study groups. LD group: 3 loading doses followed by the pro re nata regimen (3 + PRN); PRN group: 1 dose followed by the PRN regimen (1 + PRN).

The mean BCVA improved from baseline until 12 months in both groups (Figure 1(a)). At month 3, the mean change of BCVA from baseline was 8.4 letters in the PRN group and 11.9 letters in the LD group (*P*=0.157; Table 2). In the noninferior test, the PRN group was not inferior to the LD group, with a difference (PRN group minus LD group) of −3.5 letters (95% confidence interval (CI), −8.4 to 1.4; Figure 1(b)). At 1 year, the mean change of BCVA from baseline was 7.8 letters in the PRN group, compared to 10.9 letters in the LD group (*P*=0.344; Table 2). In the noninferior test, the PRN group was not inferior to the LD group, with a difference (PRN group minus LD group) of −3.1 letters (95% CI, −9.6 to 3.4; Figure 1(b)). At 1 year, PCV patients gained an average of 15.0 letters in the PRN group and 11.5 letters in the LD group (Table 3).

### 3.2. Secondary Outcomes

At month 3, the proportion of patients who did not have a decrease in visual acuity of 15 letters or more from baseline was 97.8% in the PRN group and 100% in the LD group. At 1 year, this proportion was 93.3% in the PRN group and 93.9% in the LD group. The proportion of patients who gained 15 letters or more was 31.1% in the PRN group and 38.8% in the LD group at month 3 (*P*=0.605). At 1 year, the proportion of patients gaining 15 letters or more remained nearly the same (28.9%) in the PRN group, whereas the proportion in the LD group increased to 44.9% (*P*=0.066). In total, there were no statistical differences not only in the proportion of patients who gained 15 letters or better but also in the percentage of patients who lost 15 letters or more between the two groups at 3 month and 1 year (Table 2).

**Figure 2:**
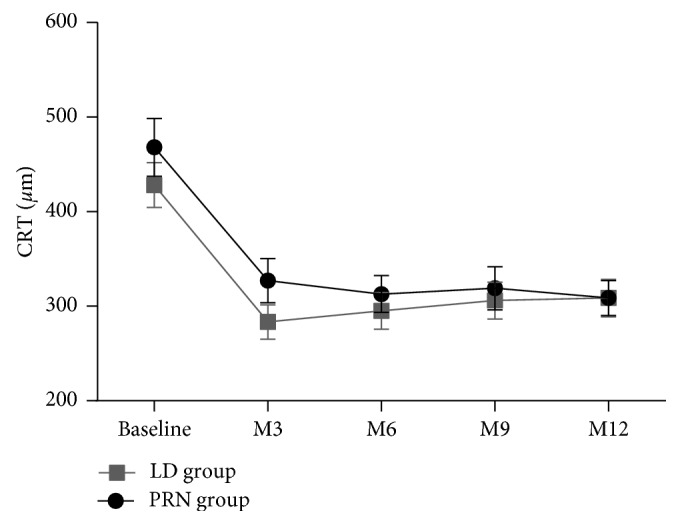
Time course of central retinal thickness (CRT) on optical coherence tomography. LD group: 3 loading doses followed by the pro re nata regimen (3 + PRN); PRN group: 1 dose followed by the PRN regimen (1 + PRN); M: month.

For anatomic end points, the mean change in CRT from baseline up to 1 year measured by OCT is presented in Figure 2. Both groups showed a significant decrease in CRT from baseline and continued through month 3. The resolution of fluid was sustained from months 3 to 12. The mean CRT reduced from 468 *μ*m at baseline to 327.2 *μ*m at month 3 and 308.7 *μ*m at month 12 in the PRN group. Meanwhile, in the LD group, the mean CRT reduced from 428 *μ*m at baseline to 283.4 *μ*m at month 3 and 308.6 *μ*m at month 12. There was no significant difference in the mean CRT reduction between two groups at month 3 (*P*=0.903) and 1 year (*P*=0.257) (Table 2).

During the first 3 months, the PRN group received an average of 2.4 injections. The number of injections differed significantly between the two groups (*P* < 0.01). Moreover, 55.6% of patients in the PRN group were treated at least three consecutive monthly injections at the beginning. At 1 year, the mean number of injections was 6.0 in the PRN group and 6.8 in the LD group, respectively. There was no statistical difference between the two groups (*P*=0.275).

### 3.3. Vision Improvement Stability

We also made a post hoc analysis for “vision improvement stability” in our patients: if the vision acuity increased by 0 or more letters after the first injection (month 1) and then decreased later by 5 letters or more compared to month 1, then we define the outcome as the “unstable effect.”

Within the first 3 months, in the PRN group, nine (20%) eyes presented reactive lesions and a decrease of 5 letters or more compared to the BCVA outcome after the first injection (month 1). While in the LD group, only one (2%) eye presented a decrease of 5 letters or more, due to retinal pigment epithelium tear. At 1 year, the proportion of the “unstable effect” was 23 of 45 eyes (51%) in the PRN group and 8 of 49 eyes (16.3%) in the LD group.

### 3.4. Safety Assessment

One patient had a stroke with minor neurological sequelae after six consecutive doses of ranibizumab injections in the PRN group who was retreated later then. One eye in the PRN group and two eyes in the LD group developed retinal pigment epithelium tear at the end of the study. No patients had any ocular SAEs, such as endophthalmitis, vitreous hemorrhage, and retinal detachment.

## 4. Discussion

In this prospective, multicenter, randomized study, we demonstrated that both PRN regimen and LD regimen using ranibizumab could improve visual acuity effectively and safely through 12 months. There were no statistical differences in CRT and visual acuity, including the mean change of BCVA and the proportion of patients with a gain or loss of 15 letters or more in BCVA between the two groups.

The results are comparable to other studies that have evaluated treatment regimens with and without loading doses. Most studies have reported the equivalent efficacy of using a PRN regimen after 1 injection compared with the PRN regimen after 3 loading dose injections. Different anti-VEGF agents have been evaluated, including bevacizumab, ranibizumab, and aflibercept [13–15]. The CATT study also suggested that a single-dose regimen has noninferior effect on functional and morphologic retinal improvement using ranibizumab or bevacizumab monthly [10]. In real world, because of the high cost and relative short half-life, there have always been discussions on the regimen optimization and the necessity of receiving three consecutive injections as loading doses [16–18]. Our data, as well as other reports, support that PRN only could provide an affordable treatment regimen with a noninferior effect on vision improvement.

Two crucial factors have been taken into consideration in PRN therapy—the interval of follow-up visits and retreatment criteria. In our study, we applied the monthly clinical visits and made the retreatment criteria according to Chinese Ocular Fundus Diseases Society in Clinical Pathway of Age-Related Macular Degeneration in China, in order to pursue the maximum stability of the lesion and visual function. In ARTIS, patients in the PRN group received mean 6 injections and patients in the LD group 6.8 injections at 1 year, which is similar to the previous studies on injection frequency, such as CATT. Moreover, the average improvement on number of letters in both groups was also similar to the previous studies on the LD regimen [19].

In our study, there was no statistically significant difference in the average improvement of CRT, the change of BCVA letters, or the percentage of eyes with a visual acuity improvement of 15 or more letters between LD group and PRN group at month 3 and month 12. In a retrospective study, Gupta et al. [14] reported the percentage of eyes with improvement of 15 letters or more at 1 year was significantly higher in the ranibizumab LD group (29.8% vs 12.9%, *P*=0.01). Menon et al. [13] also reported the percentage of patients who gained 10 or more letters was higher in the bevacizumab LD group (28%) compared with the non-LD group (26.3%), but this difference was not statistically significant.

We found that, in our cohort, the vision acuity improvement was more stable in the LD group. Within the first 3 months, in the PRN group, nine (20%) eyes presented reactive lesions and a decrease of 5 letters or more compared to the BCVA outcome after the first injection. While in the LD group, only one (2%) eye presented a decrease of 5 letters or more, due to retinal pigment epithelium tear. At 1 year, the proportion of the “unstable effect” in the PRN group was as high as 51%, which reminds us that sole PRN therapy with the current retreatment criteria based on OCT imaging may not be adequate for the prevention of the early reoccurrence of CNV in nvAMD patients. Recent ongoing OCTA researches showed that, in most nvAMD cases with the absolute resolution of fluid in or beneath the retina on OCT imaging, the area of CNV still unstably and repeatedly increased or decreased [20]. Patients receiving 3 loading doses can benefit from a more stable lesion and the less likelihood of visual outcome fluctuations. Until the coming of new biomarker for prediction of the stability of the CNV lesion, the initial 3 loading doses are indispensable in maintaining the improvement of visual outcomes.

In the current study, the subjects included are different from most other studies. PCV patients were not excluded, but as a stratification factor, and were balanced in two groups. However, the proportion of PCV included in our study (10%) was lower than the prevalence of PCV in East Asia, which may result from the inclusion criteria on the baseline visual acuity, since PCV patients were more likely to be excluded for the massive hemorrhage. On reviewing all the PCV patients included in our study at month 12, the CRT decreased in all cases, representing the distinct anatomical improvement. However, there was a discrepancy in visual outcomes-number of letters, from the 3-letter decrease to the 40-letter increase. PCV patients gained an average of 15.0 letters in the PRN group and 11.5 in the LD groups. The statistical analysis was not conducted due to the small PCV sample size. Overall, the treatment reaction and outcomes in PCV cases were similar to those in nvAMD.

The ARTIS is a multicenter, prospective, randomized, controlled clinical trial to evaluate the 12-month efficacy and safety between one dose and three loading doses followed by the PRN regimen of intravitreal ranibizumab 0.5 mg injection in treatment-naïve patients with nvAMD and PCV. The limitation is that the sample size was relatively small. Nevertheless, the results still prove that both 1 + PRN treatment of 0.5 mg ranibizumab and 3 + PRN treatment of 0.5 mg ranibizumab are of equivalent effect and safety in improving the visual acuity and reducing the CRT. However, the potential risk of the nonloading doses followed by the PRN regimen may cause early lesion recurrence and vision instability.

## Figures and Tables

**Table 1:** Baseline characteristics of the patients in two groups.

	1 + PRN	3 + PRN	*P* value
Patients (no.)	45	49	
Age (mean ± SD)	69.7 ± 8.6	70.0 ± 8.8	0.855
Male/female	30/15	30/19	0.583
Right/left eye (no.)	28/17	25/24	0.274
Letters of BCVA (mean ± SD)	49.0 ± 16.2	52.0 ± 13.9	0.341
<30 letters (no.)	8	3	0.079
30–60 letters (no.)	27	33	0.459
>60 letters (no.)	10	13	0.629
CRT (*μ*m, mean ± SD)	468 ± 202	428 ± 163	0.290
Eyes with PCV (%)	4 (8.9%)	4 (8.2%)	0.900
Pseudophakic	2	4	0.461
Patients with hypertension	10	12	0.795
Patients with diabetes	1	3	0.439
Hyperthyroidism	1	0	0.479

BCVA, best-corrected visual acuity; CRT, central retinal thickness; PCV, polypoidal choroidal vasculopathy; PRN, pro re nata; SD, standard deviation.

**Table 2:** Visual outcomes, CRT, and number of injections at month 3 and month 12.

		PRN group	LD group	*P* value
Month 3	Change of BCVA (mean ± SD)	8.4 ± 11.9	11.9 ± 11.8	0.157
	CRT (*μ*m, mean ± SD)	327.2 ± 155.0	283.4 ± 127.2	0.139
	Change in CRT from baseline	−140.8 ± 192.3	−144.7 ± 109.5	0.903
	Increase of ≥15 letters (%)	31.1%	38.8%	0.605
	Decrease of ≥15 letters (%)	2.2%	0	0.479
	Mean no. of injections	2.4	3.0	<0.01

Month 12	Change of BCVA (mean ± SD)	7.8 ± 16.0	10.9 ± 15.8	0.344
	CRT (*μ*m, mean ± SD)	308.7 ± 122.3	308.6 ± 138.6	0.999
	Change in CRT from baseline	−159.3 ± 204.2	−120.5 ± 127.1	0.257
	Increase of ≥15 letters (%)	28.9%	44.9%	0.066
	Decrease of ≥15 letters (%)	6.7%	6.1%	1.000
	Mean no. of injections	6.0	6.8	0.275

BCVA, best-corrected visual acuity; CRT, central retinal thickness; LD, loading doses; PRN, pro re nata; SD, standard deviation.

**Table 3:** Characteristics and outcomes of PCV patients.

	Group	BCVA baseline (letters)	BCVA change at month 3	BCVA change at month 12	CRT baseline (*μ*m)	CRT change at month 3	CRT change at month 12
1	PRN	69	9	15	357	−176	−164
2	PRN	69	8	8	367	−82	−112
3	PRN	72	16	15	415	−144	−201
4	PRN	67	12	22	653	−453	−441
5	LD	57	13	6	622	−342	−196
6	LD	43	34	40	413	−163	−130
7	LD	55	−3	3	457	−201	−203
8	LD	33	−9	−3	954	−126	−204

BCVA, best-corrected vision acuity; CRT, central retinal thickness; LD, loading doses; PCV, polypoidal choroidal vasculopathy; PRN, pro re nata.

## Data Availability

Access to the data used in this study needs to be permitted by Shanghai General Hospital, so they cannot be made freely available. Access to the data will be considered by the author upon request, with the permission of Shanghai General Hospital.
